# A New Reference Axis for Tibial Component Rotation in Total Knee Arthroplasty: A Three-dimensional Computed Tomography Analysis

**DOI:** 10.3389/fsurg.2022.872533

**Published:** 2022-04-27

**Authors:** Yan Jin, Pingyue Li, Yiming Yang, Xianli Zeng, Hongyuan Shen, Lihang Zhang, Tsung-Yuan Tsai, Jiarong Chen

**Affiliations:** ^1^Department of Orthopedics, PLA General Hospital of Southern Theatre Command, Guangzhou, China; ^2^Guangdong Key Lab of Orthopedic Technology and Implant, General Hospital of Southern Theater Command of PLA, The First School of Clinical Medicine, Southern Medical University, Guangzhou, China; ^3^Shanghai Key Laboratory of Orthopaedic Implants, Department of Orthopaedic Surgery, Shanghai Ninth People's Hospital, Shanghai Jiao Tong University School of Medicine; School of Biomedical Engineering, Shanghai Jiao Tong University, Shanghai, China; ^4^Engineering Research Center of Clinical Translational Digital Medicine, Ministry of Education, Beijing, China

**Keywords:** total knee arthroplasty, tibia, three-dimensional, alignment, rotation

## Abstract

The purpose of this study was to introduce a new reference axis for tibial rotation in total knee arthroplasty (TKA) and verify its reliability. A consecutive series of 80 knees that underwent TKA from 2018 to 2020 as well as 80 healthy knees were analyzed using a three-dimensional tibial model. A coordinate system was established based on the standard TKA tibial cut. The line connecting the lateral-tibial eminence and the medial 1/3rd of the tibial tubercle or the medial border of the tibial tubercle was identified as the lateral eminence line (LE line) and the medial lateral eminence line (MLE line), respectively. To evaluate the reliability of the new reference axis, Akagi's line, the medial third of the tibial tubercle (1/3 line) was compared with the LE and MLE lines by measuring the angle between the lines and the Z-axis. In the coronal view, the intersection angle (TPA), which is composed of the line connecting the center of the medial and lateral tibial plateau with the Z-axis, was measured. The mean angle between Akagi's line and the Z-axis in the healthy group and the osteoarthritis (OA) group was 87.57 ± 3.48° and 87.61 ± 3.47°, respectively. The mean angle between the LE line and Z-axis in the healthy and OA groups was 87.15 ± 4.13° and 86.78 ± 3.95°, respectively. A weak correlation was found between the TPA and Akagi's line and the 1/3 line. A moderate correlation was observed between the TPA and LE lines. There were no significant differences between the healthy and OA groups (*P* > 0.05) in any of the four reference axes. The LE line showed excellent intra- and inter-observer reliability and reproducibility. The novel and easily drawn LE line is a preferable option for tibial component rotational alignment in TKA.

## Introduction

In total knee arthroplasty (TKA), the rotational alignment of the tibial component plays an important role in the axial plane. Malrotation of the tibial component may lead to premature wear of the polyethylene liner, abnormal patellar tracking, anterior knee pain, aseptic loosening, knee stiffness, and other complications (1–4).

The trans-epicondylar axis (TEA) is a well-recognized anatomic reference axis for evaluating the rotational alignment of the femoral prosthesis. Theoretically, the line perpendicular to the projected TEA on the tibia can be used to evaluate tibial rotational positioning in TKA. However, it is difficult to project the TEA onto the tibial plateau during TKA, and the assumed line cannot be marked intraoperatively. The most reliable anatomical reference axis for tibial rotational alignment remains controversial (5–10). Various reference axes have been proposed to aid component alignment, including Akagi's line (5) and the medial third of the tibial tubercle (1/3 line) (11). However, these methods have shortcomings, including osteophyte influence (12) and excessive external rotation (13); moreover, if a two-dimensional (2D) measurement method is used, the accuracy of measurement might be affected by the layer thickness and scan spacing (14).

Based on our clinical experience, a new reference axis that would pass through the lateral eminence of the tibia and the medial third of the tibial tubercle was designed. The aim of our study was to verify the reliability of the new reference axis of tibial rotation in total knee arthroplasty (TKA).

## Methods

### Study Population

Eighty healthy volunteers and 80 patients with varus OA were included in this study. The inclusion criteria were as follows: (i) patients who had been diagnosed with knee osteoarthritis; (ii) patients who had undergone CT scans of the entire lower extremity and the quality of the data was good enough for three-dimensional (3D) reconstruction; (iii) regarding the staging of knee osteoarthritis, patients had Kellgren–Lawrence stage II to IV with intact tibial plateau posterior without bone defects. The exclusion criteria were as follows: (i) previous lower extremity surgery; (ii) obvious valgus deformity, when the knee was upright and close together, the distance between the medial malleolus was more than 3 cm; (iii) any immunological or endocrine diseases of the knee; (iv) severe osteoporosis; and (v) osteofusion of the knee joint. The risk of radiographic exposure during a computed tomography (CT) scan was informed to the volunteers, and informed consent forms were signed. The study was approved by the hospital's institutional review board.

### Image Acquisition and 3D Model Reconstruction

Transverse CT scans (Somatom Sensation 64, Siemens Inc., Munich, Germany) of the subjects' full-length lower limbs were captured at high resolution (521 × 521, thickness = 0.8 mm; pitch = 1). All images and dates were saved as Digital Imaging and Communications in Medicine (DICOM) files on a personal computer. During the scanning, the knee position was kept neutral, and the scans were performed perpendicular to the lower limb. The same protocol was followed for all the CT scans. Three-dimensional reconstructions were performed from the CT data using Mimics 19.0 (Materialize, Leuven, Belgium). After the removal of osteophytes, 3D models were imported into Rhinoceros 5.0 (Robert McNeel & Assoc, USA), and the morphology was measured in Rhinoceros.

### Establishment of the Coordinate Axis

**Figure 1:**
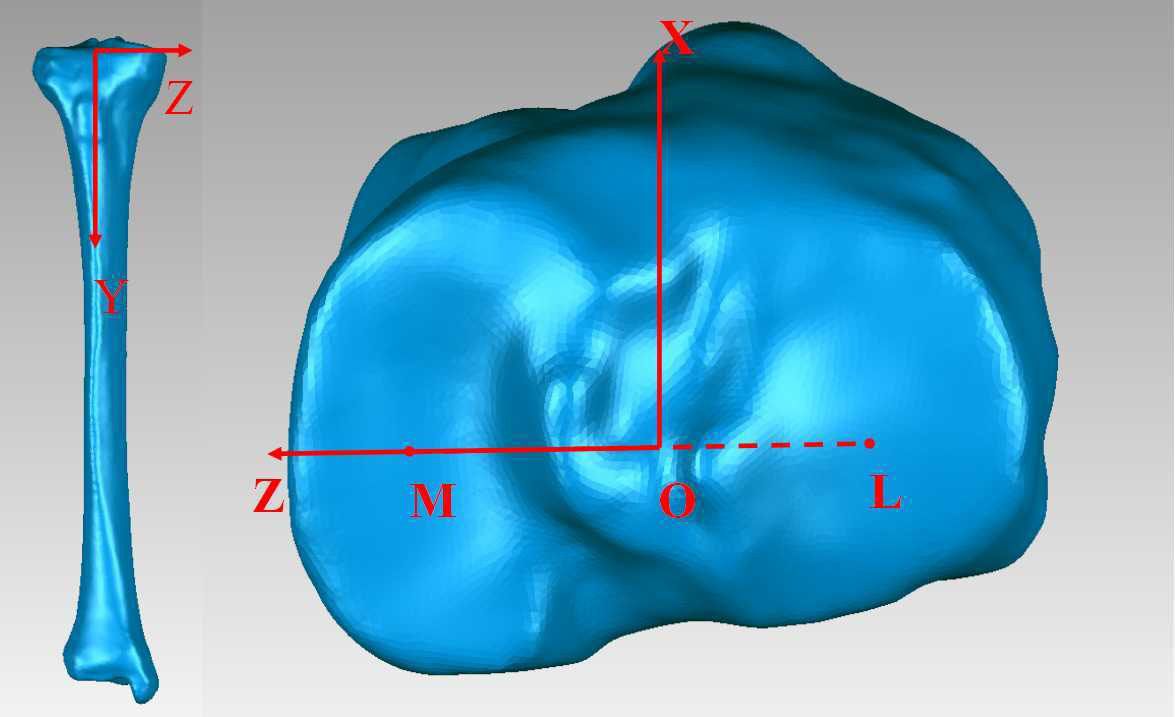
M, the center of the medial tibial plateau; L, the center of the lateral tibial plateau; O, the origin of the coordinate system.

Some patients with OA with flexion contracture cannot fully extend the knee, and the projection of the TEA is greatly affected. A coordinate system based on a standard TKA tibial cut was established as the reference. All measurements and simulations were based on this coordinate system (Figure 1). The center of the medial and lateral tibial plateaus was recognized, and the line connecting the two points was defined as the M-L line. The coordinate origin was defined as the midpoint (O) of the M-L line (6). The Y-axis was defined as the line best fitting the centroid of the tibial canal 5–15 cm from the tibial tubercle. The X-axis line was defined as a line perpendicular to the M-L line and passed through the O point. The Z-axis was defined as the line perpendicular to both the X-axis and the Y-axis.

### Confirming Lines and Outcome Measurement

To stimulate the operation, the center of the posterior cruciate ligament (P) and the lateral tibial eminence (L) were projected on the axial plane along the Y-axis. The medial border and medial 1/3 of the tibial tubercle were identified and projected onto the plane. The line connecting the P point and medial border of the tibial tubercle was defined as Akagi's line (5), while the 1/3 line was defined as the line connecting the P point and 1/3 of the tibial tubercle (11). The line connecting the L point and the medial 1/3 of the tibial tubercle was identified as the lateral eminence line (LE line). The line connecting the L point and the medial border of the tibial tubercle was defined as the medial lateral eminence (MLE) line.

**Figure 2:**
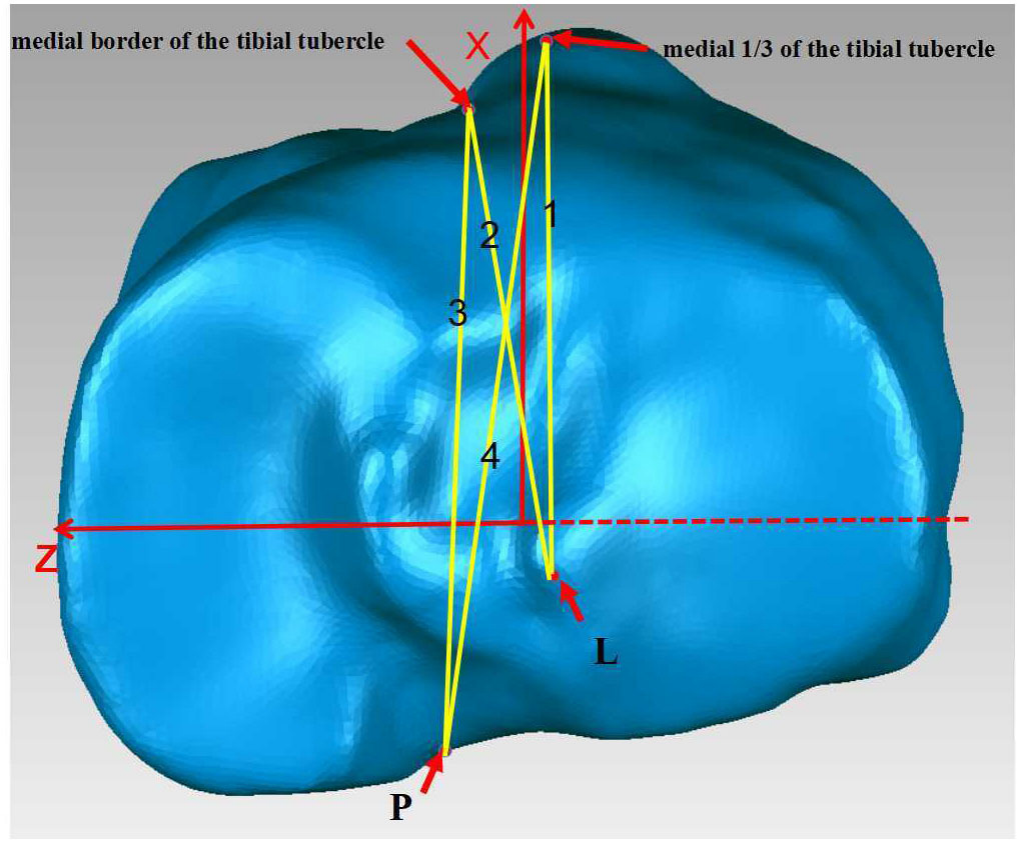
1.LE line, 2.MLE line, 3.Akagi's line, 4.1/3 line.

**Figure 3:**
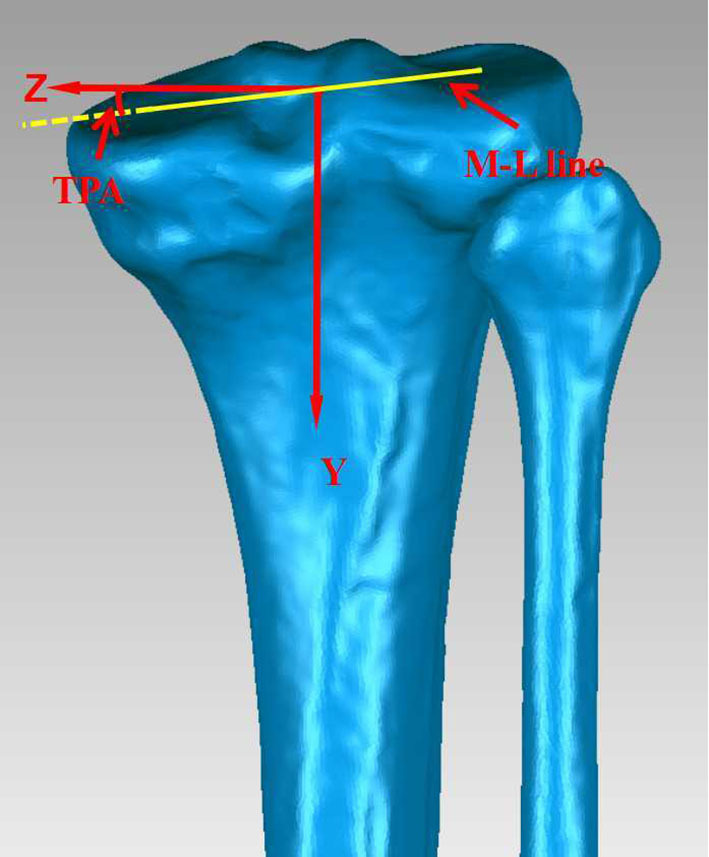
Definition of the TPA: the intersection angle of the M-L line with the Z-axis.

With the section plane perpendicular to the sight, the angles of Akagi's line, 1/3 line, MLE line, and LE line with the Z-axis were measured (Figure 2). In the coronal view, the intersection angle (TPA) of the M-L line with the Z-axis was acquired and used to evaluate the internal inclination of the tibial plateau (Figure 3). Measurements of the angles and distances were performed by one observer, and intraobserver variations in the measurements were assessed by repeating the process 10 times, with an interval of 2 weeks between two measurements. The mean of the data was saved as the true value. Two sample *t*-tests were performed to test for differences between the groups.

### Statistical Analysis

Student's *t*-test was performed to test the differences between Akagi's line, the 1/3 line, and the LE line. Pearson's correlation analysis was performed to evaluate the associations between various angles. *P* < 0.05 were considered to be statistically significant. All statistical analyses were performed using SPSS 24.0 (IBM, Armonk, New York, USA).

## Results

**Figure 4:**
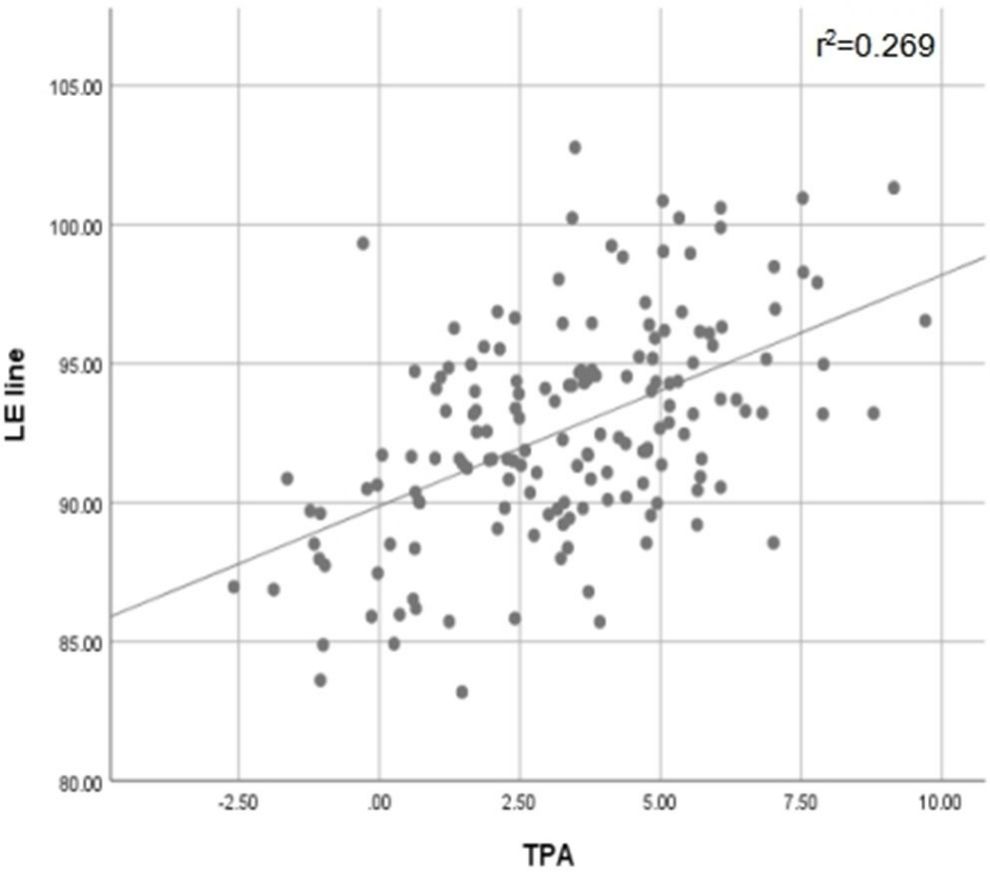
Scatter Plots of LE line and TPA with Pearson's correlation test. Correlation level (absolute value): low correlation (< 0.3); moderate correlation (≥0.3 to < 0.5); high correlation (0.5 < to < 1). r^2^, coefficient of determination.

The mean hip-knee-ankle (HKA) angles of the healthy and OA groups were 179.8 ± 2.02 and 176.12 ± 4.63, respectively. The mean angles between Akagi's line and the Z-axis in the healthy and OA groups were 92.65 ± 3.45 and 92.16 ± 3.30, respectively. The mean angles between the 1/3 line and Z-axis in the healthy and OA groups were 98.82 ± 3.18 and 98.33 ± 3.16, respectively. The values for the mean angle between the LE line and Z-axis in the healthy and OA groups were 92.38 ± 4.05 and 92.95 ± 3.67, respectively. The values for the mean angle between the MLE line and Z-axis in the healthy and OA groups were 82.92 ± 4.77 and 83.83 ± 4.47, respectively (Table 1). There were no significant differences between the healthy and OA groups (*P* > 0.05) in any of the four reference axes. A significant difference was found between the 1/3, LE, and MLE lines (*P* < 0.01). A significant difference was found between the LE line and 1/3 line as well as between the MLE and Akagi's lines and the 1/3 line. There were no significant differences between Akagi's line and the LE line. The mean TPA values of the healthy and OA group were 3.20 ± 2.04 and 3.49 ± 2.81, respectively. There was no difference between the TPA of the OA group and that of the healthy group. A high correlation (0.519) was found between the TPA and the mean angle between the LE line and the Z-axis (Figure 4).

**Table 1:** Comparison of the OA group and healthy group.

	**OA group**	**Healthy group**	***P*-value**
Akagi's line	92.16 ± 3.30°	92.65 ± 3.45°	0.36
1/3 line	98.33 ± 3.16^°#^	98.82 ± 3.18^°#^	0.32
LE line	92.95 ± 3.67^°Δ^	92.38 ± 4.05^°Δ^	0.35
MLE line	83.83 ± 4.47^°*#Δ^	82.92 ± 4.77^°*#Δ^	0.21

*^#^Means significant difference was found with Akagi's line (P < 0.01)*.

*^Δ^Means significant difference was found with 1/3 line (P < 0.01)*.

*Means significant difference was found with LE line (P < 0.01).

## Discussion

The results of the current study showed that most of the LE lines were located between Akagi's line and the 1/3 line. Our results showed that most of the LE lines were located between Akagi's line and the 1/3 line. The result of Pearson's correlation between the TPA and LE lines showed a significant correlation. Increasing TPA results in a greater intersection angle; this indicates that the increase in varus deformity might lead to an increase in the external rotation of the proximal tibia. This is consistent with the results of a previous study (15). The greater the varus of the tibia, the closer the anterior landmark would be to the medial border of the tibial tubercle. This indicates that the LE line might be better for patients with OA with deformity or moderate varus deformity. When the TPA was greater than 5.87, the MLE line was closer to 90 than the other axes. We, therefore, speculate that the MLE line was much better than the other axes when the TPA was too large. However, further studies are required.

Various reference axes have been proposed to achieve ideal tibial rotational alignment. Akagi's line and the medial third of the tibial tubercle (1/3 line) have been the most commonly used. The LE line, which connects the lateral tibial eminence and medial 1/3 of the tibial tubercle, was first proposed in our study. The reasons for choosing the LE line as the reference axis for tibial rotation are as follows. First, the lateral eminence of the tibia is much easier to identify (6). As reported by Cobb et al. the tip of the lateral tibial eminence is an identifiable and reliable posterior bony landmark. Second, the medial third of the tibial tubercle was reported to be better than the medial border of the tibial tubercle for tibial rotational alignment (8). Third, the LE line showed no significant difference between the OA and healthy groups, which proves the stability of the LE line.

The reliability and stability of Akagi's line have been documented in previous studies (8, 9, 13, 16, 17). Akagi et al. (5) measured the angle between the line perpendicular to the projected surgical transepicondylar axis (SEA) and Akagi's line. He found that the mean angle between them was 0.0 ± 2.8 (range: −6.3 ± 5.2), which was significantly better than that between the 1/3 line (10.0 ± 4.2 vs. 1.6–19.5). Akagi et al. (16) compared the Akagi line with the transmalleolar axis and the second metatarsal bone axis in 57 healthy adults. They found that the mean angles between the line perpendicular to SEA and Akagi's line were −0.2 ± 2.8 (−5.5 to 6.3), and those between the SEA and the transmalleolar axis and second metatarsal bone axis were 25.9 ± 9 (8–49.4) and 5.2 ± 10 (−21.9 to 24), respectively. Similarly, in the present study, the mean angle of Akagi's line and the Z-axis was much closer to 90 than the other axes, which showed good reliability of Akagi's line. Furthermore, there was no significant difference between Akagi's line and the LE line, both in the OA and healthy groups, indicating that the LE line was as reliable as Akagi's line. However, Kawahara et al. (18) reported that it is difficult to recognize the midpoint of the PCL after resection of the proximal tibia during surgery. In particular, the PCL was completely removed with the posterior stabilized prostheses, and the midpoint of the PCL would be difficult to recognize. Compared to the midpoint of the PCL, the lateral eminence of the tibia was much easier to identify, and wear of the lateral eminence of the tibia was rare in OA patients. The tibial tubercle is an easily recognizable anatomical landmark, and a previous study has demonstrated its reliability (8). Hence, the LE line was much easier to identify intraoperatively than Akagi's line.

The 1/3 line has been recommended by some authors (19, 20). Wernecke et al. (21) performed MRI of 544 cases of normal knee joints and believed that the 1/3 line was a reliable landmark for the rotational alignment of the tibial component, which may optimize femorotibial kinematics in fixed-bearing TKA. However, some studies have reported that this might lead to excessive external rotation of the tibial component (13). Our results indicated that the 1/3 line would lead to excessive external rotation of the tibial component, which is consistent with the results of some previous studies (13, 16, 22, 23). Thirty-two cases of normal knee joints were reconstructed by Yang et al. (13); they measured the angles between the 1/3 line and the projected surgical transepicondylar axis (STEA) and clinical transepicondylar axis (CTEA). They found the mean angle between the STEA and CTEA were 102.4 ± 2.7 (95.3–106.5) and 106.9 ± 2.9 (98.4–112.6), respectively. Compared to the 1/3 line, the mean angle of the LE line and Z-axis was much closer to 90 (92.95 vs. 98.33). This could help avoid excessive external rotation of the tibial component in TKA by using the LE line as a reference axis. Other reference axes have been reported in previous studies. The PC line was defined as a line perpendicular to the tibial posterior condyle (12). However, the identification of the PC line is affected by osteophytes or bony defects of the posterior tibial condyles (12). The midline was defined as a line passing through a point 1 mm medial to the medial border of the tibial tubercle to the midsulcus of the tibial eminence (24). It was difficult to recognize the midsulcus of the tibial eminence through traditional imaging methods (13) in patients with OA, whose tibial eminence was worn (22).

This study has some limitations. First, we excluded patients with valgus deformities. Second, only Chinese participants were included in the study. There may be anatomical differences between the Caucasian and Chinese populations. Third, further research is required to prove that the MLE line is better than other axes.

In summary, the LE lines studied showed excellent intra- and inter-observer reliability and reproducibility. The novel and easily drawn LE line showed a strong correlation with Akagi's line and can be used for tibial component rotational alignment in TKA.

## Data Availability Statement

The raw data supporting the conclusions of this article will be made available by the authors, without undue reservation.

## Ethics Statement

The studies involving human participants were reviewed and approved by PLA General Hospital of Southern Theatre Command: People's Liberation Army General Hospital of Southern Theatre Command. The patients/participants provided their written informed consent to participate in this study.

## Author Contributions

The first draft of the manuscript was written by YJ. Data collection and analysis were performed by YY. Interpretation of data was performed by XZ, LZ, HS, PL, JC, and T-YT had substantively revised it. All authors contributed to the study, collected data, read, and approved the final manuscript.

## Funding

This project was sponsored by the National Natural Science Foundation of China (81871808).

## Conflict of Interest

The authors declare that the research was conducted in the absence of any commercial or financial relationships that could be construed as a potential conflict of interest.

## Publisher's Note

All claims expressed in this article are solely those of the authors and do not necessarily represent those of their affiliated organizations, or those of the publisher, the editors and the reviewers. Any product that may be evaluated in this article, or claim that may be made by its manufacturer, is not guaranteed or endorsed by the publisher.
